# Adaptive load feedback robustly signals force dynamics in robotic model of *Carausius morosus* stepping

**DOI:** 10.3389/fnbot.2023.1125171

**Published:** 2023-01-26

**Authors:** William P. Zyhowski, Sasha N. Zill, Nicholas S. Szczecinski

**Affiliations:** ^1^Department of Mechanical and Aerospace Engineering, West Virginia University, Morgantown, WV, United States; ^2^Department of Biomedical Sciences, Marshall University, Huntington, WV, United States

**Keywords:** campaniform sensilla, dynamic scaling, insects, legged locomotion, robotics, strain gauges

## Abstract

Animals utilize a number of neuronal systems to produce locomotion. One type of sensory organ that contributes in insects is the campaniform sensillum (CS) that measures the load on their legs. Groups of the receptors are found on high stress regions of the leg exoskeleton and they have significant effects in adapting walking behavior. Recording from these sensors in freely moving animals is limited by technical constraints. To better understand the load feedback signaled by CS to the nervous system, we have constructed a dynamically scaled robotic model of the *Carausius morosus* stick insect middle leg. The leg steps on a treadmill and supports weight during stance to simulate body weight. Strain gauges were mounted in the same positions and orientations as four key CS groups (Groups 3, 4, 6B, and 6A). Continuous data from the strain gauges were processed through a previously published dynamic computational model of CS discharge. Our experiments suggest that under different stepping conditions (e.g., changing “body” weight, phasic load stimuli, slipping foot), the CS sensory discharge robustly signals increases in force, such as at the beginning of stance, and decreases in force, such as at the end of stance or when the foot slips. Such signals would be crucial for an insect or robot to maintain intra- and inter-leg coordination while walking over extreme terrain.

## 1. Introduction

Insects are useful models for the design and control of walking robots. Insects walk on a variety of substrates, at a variety of orientations, and at various speeds with little apparent effort. A robot that could walk as capably as an insect would have applications in fields such as agriculture, mining, forestry, and search-and-rescue. However, despite how much is known about how insects control their locomotion, several components of their sensorimotor systems are not fully understood. One such component is the campaniform sensillum (CS), a strain-sensing organ embedded into the exoskeleton cuticle (Pringle, 1938; Spinola and Chapman, 1975). Insects possess several such sensors in parts of the leg that are subjected to high bending loads (Pearson and Iles, 1973; Harris et al., 2020). CS produce highly dynamic discharge patterns in response to leg segment strain, with many adapting in response to tonic forces. Recordings from CS in standing and walking animals reveal that CS are capable of encoding many features of the force acting on the leg, most notably the rate of change of the strain (Noah et al., 2004; Keller et al., 2007). However, due to the difficulty of recording from multiple groups of CS simultaneously, it is difficult to conceptualize how multiple groups across the leg respond to forces imposed on the leg.

To address this challenge, we have developed a robotic leg that has incorporated sense organs and mechanisms of force feedback found in walking of the stick insect, *Carausius morosus* (*C. morosus*). This study shows that complex and adaptive signals from the biological receptors CS can be emulated using simple mechanical sensors (strain gauges) whose output is processed through a mathematical model of the receptors (Szczecinski et al., 2021). These mechanisms effectively signal phase changes and variations in body load (as occur in gait) and provide a sensitive measure for detection of decreases in substrate adhesion and leg slipping. This robotic model is a platform for conducting biological experiments that would be difficult to perform in the animal.

### 1.1. Function of campaniform sensilla

In the legs of animals, modest forces are generated by muscles to move the legs relative to the animal’s body (as when a leg is lifted in the swing phase of walking). Much larger forces are produced when the legs contact the ground during the stance phase and skeletal muscles act to move the animal’s body relative to the substrate. In insects, forces are detected by CS, receptors that monitor strains in the exoskeleton. Previous studies have shown that CS discharges in walking occur (almost entirely) during the stance phase when muscles contract against a resistance. CS can then function as proprioceptive sense organs monitoring the animal’s own behaviors by detecting the net effect of resisted muscle forces and variations in the effects of body load due to gaits. These signals are used to adjust the magnitude of muscle contractions and timing of phase transitions in walking (Zill et al., 2004; Buschmann et al., 2015).

Many groups of CS are located on the exoskeleton close to the insertions of leg muscles. In these locations, the strains in the exoskeleton can be calculated as functions of the joint torques, the net rotational forces that occur about the joint (independent of the joint angles) due to muscle contractions and loads (Zill et al., 2021). Recent studies have examined the signals produced by the tibial group of CS to joint torques calculated by inverse dynamics. These data are obtained from stick insects walking freely on horizontal or sloped surfaces (Dallmann et al., 2016, 2019; Zill et al., 2018; Harris et al., 2022). These studies have shown that the sensory discharge in response to these “naturalistic” stimuli are dominated by the dynamic sensitivities of the receptors, enabling them to precisely monitor variation in forces (Dallmann et al., 2016, 2019; Zill et al., 2018; Harris et al., 2022), which previous studies have termed “yank” (Lin et al., 2019). These sensitivities could contribute to ongoing modulation of muscle contractions to generate the smooth and graded movements characteristic of walking in animals (Günzel et al., 2022).

### 1.2. Previous robot models

Biomimetic robots have been used for decades to better understand the mechanisms underlying motor control of insects and other animals (for a review, see Manoonpong et al., 2021). Robotic models of animals complement purely computational models in that they must confront the full physics of the behavior being modeled. In the present study, we are interested in measuring the minute strain of the leg segments as the leg steps, but to reduce runtime, multi-body physics simulators almost always model segments as “rigid bodies” that cannot bend. Computational modeling techniques such as Finite Element Analysis (FEA) are extremely useful for predicting how complex shapes such as insect leg segments would strain when stressed and have yielded valuable insights into how insects detect strain (Kaliyamoorthy et al., 2001; Wang et al., 2014, 2019; Noda et al., 2018; Dinges et al., 2022). However, determining and applying a realistic stress profile to the model is a challenging problem, which a robotic model inherently solves. Furthermore, phenomena such as ground contact forces, including static and sliding friction, are critical to determining the load on each leg segment, but are notoriously difficult to model accurately (Greenwood, 1988; Khalil, 2002). By running the robotic leg’s strain measurements through our dynamic model of CS discharge, we effectively embody the CS model and offer a glimpse into the sensory feedback the animal may receive during walking.

Robotic models of animal legs also have the advantage that they can be leveraged to produce freely walking robots that apply biological principles directly to the control of locomotion. To explore how load feedback contributed to the coordination of the joints throughout one leg, a neuromechanical model of insect walking (Ekeberg et al., 2004) was applied to produce the SCASM (Sensory Coupled Action Switching Modules) single-leg stepping controller (Lewinger et al., 2006) which was later incorporated into Bill-Ant (Lewinger and Quinn, 2010). Similarly, the same neuromechanical model was applied to produce the robot Octavio (von Twickel et al., 2012) whose single leg and whole body were used to test biologically based and evolved control networks (von Twickel et al., 2011, 2012). Both these single-leg robotic models were simplifications of the full dynamics of a walking animal. However, each single-leg model enabled the researchers to thoroughly test how the structure and tuning of the control system generated reliable stepping without the added complications caused by additional legs. Single-leg models may be viewed as prototypes for more complete, future robots that more thoroughly test the biological principles that underlie locomotion. Progressions such as these support the use of single leg robotic models to better understand the control of walking in animals and robots.

Multiple recent robots incorporate strain gauges in their legs to mimic CS, including Hector (Dürr et al., 2019), Mantisbot (Szczecinski et al., 2015), and Drosophibot (Goldsmith et al., 2020). These robots were built as biomimetic models of the stick insect, praying mantis, and fruit fly, respectively. These robots could be used to predict what sensory discharge the CS produce during walking. However, to the authors’ knowledge, none of these robots have been used for this purpose. Furthermore, to the authors’ knowledge, none of these robots except Drosophibot filter strain gauge feedback to mimic the dynamics of CS, but this feature was only tested in simulation. As a result, the present study is novel in that it is the first robotic model of an insect leg in which CS sensory discharge is predicted by processing strain gauge readings in an animal-like way.

In this study we investigate how the nervous system of an insect may experience dynamic load feedback. We monitor the strain and calculate corresponding CS discharge from Groups 3, 4, 6A, and 6B as the robotic leg steps on a treadmill and supports some of its body weight. To validate that the model captures the basic discharge properties of CS, we first reproduce biological experiments such as responses to ramp and hold stimuli in different orientations (Zill et al., 2012). We hypothesize that the geometry of the leg, coupled with the directional sensitivity of CS and strain gauges, will affect the amplitude of sensory discharge at any particular moment. Furthermore, we expect that the dynamical features of the CS model will emphasize changes in load, whether due to the onset of stance, end of stance, addition of weight throughout stance, or foot slipping. We discuss implications for the control of walking in both insects and robots. Preliminary results from some of these experiments were published at the Living Machines conference (Zyhowski et al., 2022).

## 2. Materials and methods

### 2.1. Robotic leg construction and control

**FIGURE 1:**
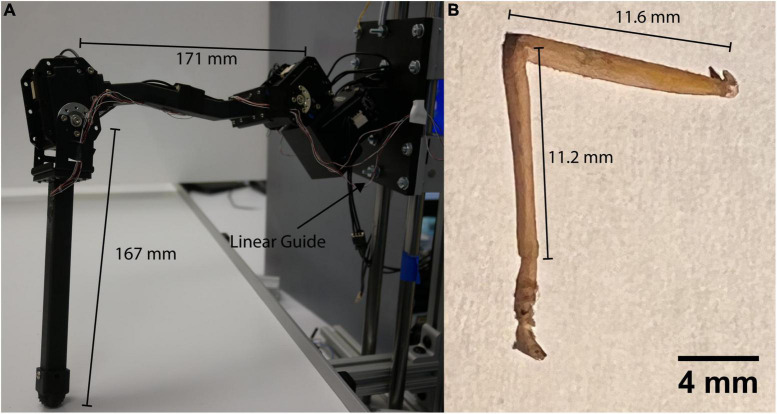
**(A)** *C. morosus* robotic model of the middle leg connected to a linear guide. The linear guide is constrained to movement in the vertical direction or z axis. The free movement in the vertical directions forces the leg to support its own weight as it steps on the treadmill. **(B)** Middle leg of a *C. morosus* for biological size and degrees of freedom comparison.

The robotic leg in Figure 1 is comprised of three MX-28AT Dynamixel servomotors and 3D printed parts (MX-28 e-Manual, 2023). The servomotors are connected in series by custom-designed brackets intended to apply balanced loads to each servomotor shaft. This is particularly important as it allows strain to be concentrated at CS sites as it is in the animal. The leg is a 15:1 scale model with the same segmental proportions as the middle leg of a *C. morosus* stick insect (Cruse and Bartling, 1995; Theunissen et al., 2015). Each leg segment is a hollow square tube with external dimensions 1 cm by 1 cm and a wall thickness of 1 mm. The tubes were printed from Onyx (Mark Two, 2022) with a Markforged Mark II 3D printer (Composites, 2022). Using hollow tubes for construction facilitates comparison to the stress and strain experienced by insect exoskeletons, which can be approximated as hollow tubes (Zill and Moran, 1981).

**FIGURE 2:**
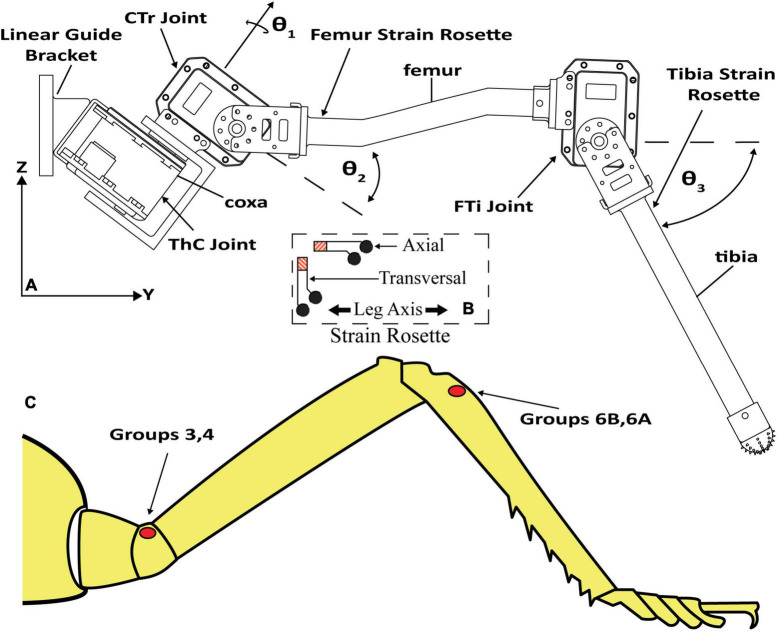
**(A)** *C. morosus* robotic middle leg with three degrees of freedom. The leg segments, joint axes, and joint angles are indicated. The strain gauge rosettes are labeled on the tibia and trochanterofemur. **(B)** Strain gauge rosette inset, which displays orientation, is relative to the long axis of the leg segment. **(C)** CS groups which are labeled on an illustration of a *C. morosus* leg.

Figure 2 displays the leg with the thorax-coxa (ThC), coxa-trochanter (CTr), and femur-tibia (FTi) joints labeled. The ThC servomotor is mounted to a carriage that is free to slide along a vertically oriented linear guide. The carriage simulates the movement and mass of the insect body. Two strain gauge rosettes capture the strain data which measures the transversal and axial strain of each leg segment. One is mounted to the proximal dorsal face of the trochanterofemur, and one is mounted to the proximal dorsal face of the tibia. The locations and orientations of the rosettes are comparable to the locations and orientations of major CS groups 3, 4, 6A, and 6B (Delcomyn et al., 1996; Ridgel et al., 2001).

**FIGURE 3:**
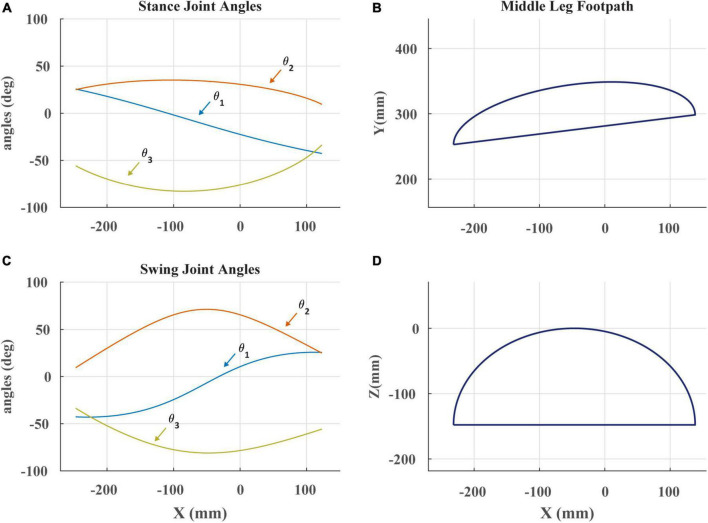
**(A)** Stance angles of robotic leg plotted against the x-coordinate of the foot in space as in Cruse and Bartling (1995). The x-coordinate is the anterior-posterior position of the foot. θ_1_ is the ThC joint, θ_2_ is the CTr joint, and θ_3_ is the FTi joint. **(B)** The projected footpath of the scaled *C. morosus* footpath in the x-y plane. The y-coordinate is the lateral position of the foot. **(C)** Swing joint angles of robotic leg plotted against the x-coordinate of the foot in space. **(D)** The projected footpath on the x-z plane. The z-coordinate is the dorso-ventral position of the foot.

A desktop computer running a MATLAB script was utilized to command servomotor angles. These angles were calculated using inverse kinematics to follow the desired footpath (Figure 3). The angles were then sent over serial communication to an OpenCM 9.04 microcontroller acting as an intermediary that then passed them onto the MX-28AT Dynamixel servomotors. The serial bus was running at 60 samples per second. The OpenCM was also utilized to collect the analog strain data and relayed it back to the MATLAB script. This was done through the OpenCM’s onboard 12-bit analog to digital convertor (ADC).

### 2.2. Robot forward and inverse kinematics

The leg was designed to contain the same degrees of freedom as the *C. morosus* middle leg (Cruse and Bartling, 1995; Theunissen et al., 2015; Zyhowski et al., 2022). The forward kinematics of the leg can be formulated as a chain of homogenous transformation matrices, each assembled using the product of exponentials formula [Equation (1)]. In our leg, θ_1_ is the angle of the ThC joint, θ_2_ is the angle of the CTr joint, and θ_3_ is the angle of the FTi joint (Lynch and Park, 2017):


(1) $$g_{d}\left(\theta_{1},\theta_{2},\theta_{3}\right)=e^{{\hat{\xi }}_{1}\theta_{1}}e^{{\hat{\xi }}_{2}\theta_{2}}e^{{\hat{\xi }}_{3}\theta_{3}}g_{st}$$

The 4 × 4 matrix *g_d_*, expanded in Equation (2), describes the configuration of the foot relative to the body. The upper-left 3 × 3 block of the matrix, *R*, describes the rotation of the foot in space; the upper-right 3 × 1 vector, *p*, describes the position of the foot in space; the lower-left 1 × 3 vector is all 0; and the lower-right element is 1:


(2) $$g_{d}\left(\theta_{1},\theta_{2},\theta_{3}\right)=\left[\begin{array}{cc} R\left(\theta_{1},\theta_{2},\theta_{3}\right) & p\left(\theta_{1},\theta_{2},\theta_{3}\right)\\ 0 & 1 \end{array}\right]$$

Each matrix exponential $e^{{\hat{\xi }}_{\text{i}}\theta_{\text{i}}}$ is composed in the same way but with joint-specific joint twists, ξ_*i*_, which are listed in Table 1. The constant matrix *g*_*st*_ follows this same arrangement and holds the state of the end effector when all three joint angles are equal to 0 (i.e., the zero configuration). Please see Lynch and Park (2017) for more details on how to formulate forward kinematics in this way.

**TABLE 1:** Zero configuration of model parameters in vector form.

Zero configuration vectors			
	x	y	z
ω_1_	0	−sin (37^o^)	−cos (37^o^)
ω_2_	1	0	0
ω_3_	1	0	0
*q*_1_	0	61.34	15.03
*q*_2_	0	93.46	22.36
*q*_3_	0	230.45	−80.90
*q*_*end*_	0	362.42	−180.99

The direction of twist is denoted with ω, and the axis of rotation is denoted with q (mm).

To place the foot at a particular point in 3D space, the angles θ_1_, θ_2_, and θ_3_ must be calculated (i.e., the inverse kinematics problem). Because this inverse kinematics problem requires that we calculate 3 joint angles to place the foot in a particular 3D point in space, there is a unique set of joint angles for every foot position. This means that once a footpath (i.e., sequence of foot positions over time) was established, the joint angles could be uniquely specified. There are many ways to solve this problem. We solved this problem by defining the function $\vec{f}$ in Equation (3) and using a quasi-Newton method [fsolve in Matlab. (2021)] to find θ_1_, θ_2_, and θ_3_ that satisfy $\vec{f}=\vec{0}$,


(3) $$\vec{f}\left(\theta_{1},\theta_{2},\theta_{3}\right)=\vec{p}\left(\theta_{1},\theta_{2},\theta_{3}\right)-{\left[x_{desired},y_{desired},z_{desired}\right]}^{T}=\vec{0}.$$

Footpaths seen in Figure 3 were modeled after those of *C. morosus* (Cruse and Bartling, 1995). This was done by constructing a series of piecewise polynomials to create the appropriate trajectory shape. Care was taken to ensure that when the robot’s tarsus was lowered to the treadmill, the tarsus’s velocity matched that of the treadmill. This required that at the end of swing, the tarsus overshoot the location of touchdown, accelerate rearward until its speed matched the treadmill, then lower to contact the treadmill. Failure to match the tarsus and treadmill speed resulted in “bouncing” of the leg at the beginning of stance that is not observed in walking stick insects (Zyhowski et al., 2022). This likely occurs because the robotic leg has no compliant tarsus, but instead presses its relatively rigid tibia into the treadmill. Adding a compliant tarsus to the robotic leg in the future may resolve this issue.

### 2.3. Strain gauges and data

Like many sensors, strain gauges transduce strain as a change in resistance. When implemented, this results in a small change in the voltage drop across the sensor. Most ADCs, including the OpenCM’s ADC, cannot read such small signals. A differential operational amplifier (Op-Amp) was employed to amplify the signal by a designated gain of *250* which is enough gain for the ADC to function. This Op-Amp has manually adjustable trimpots to adjust the offset, which can change over time. This is due to a variety of reasons such as the gauges themselves are susceptible to temperature fluctuations.

Strain data was filtered by a moving median filter with a window of 13 timesteps. This filter removed single-step fluctuations in the data due to electrical noise or ADC problems without affecting the strain profile over time. This filter enabled reliable collection of strain data that could be directly run through the CS model to simulate their sensory discharge.

### 2.4. Campaniform sensilla (CS) sensory discharge model

Campaniform sensilla produce adaptive sensory discharge in response to leg bending. Although the discharge of the smaller amplitude CS reflects the tonic bending moment in extracellular recordings (Zill et al., 2004), the peak discharge of the large amplitude CS is dominated by a power-law encoding of the rate of change of leg bending (Ridgel et al., 2000). The large amplitude CS exhibit further dynamical features such as discharge adaptation in response to tonic bending (Zill and Moran, 1981), hysteresis under cyclic bending (Noah et al., 2001, 2004), and phasic activation in some groups when bending decreases rapidly (Keller et al., 2007; Zill et al., 2009). The dynamic responses of CS are hypothesized to contribute to the adaptive nature of insect locomotion, including the ability to detect when the load on the leg increases suddenly due to gait or external factors (Ridgel et al., 2000) or decreases suddenly due to foot slipping (Harris et al., 2022).

Previously, a simple non-linear phenomenological model was developed in which one adaptive mechanism could explain all the previously mentioned dynamic responses (Szczecinski et al., 2021). Specifically, the output of the model *y* is dominated by the bending load *u relative to* an adaptive threshold *x* that follows the instantaneous bending load:


(4) ymax⁡(0,a⋅(u-x)+c⋅u+d)

(5) $$\tau \cdot \dot{x}\text{sign}\left(u-x\right)\cdot {\left|u-x\right|}^{b}$$

where *a, b, c, d*, and τ are constant parameters that are tuned to reproduce the dynamics of sensory recordings from animals. Previous work has shown that the model generalizes well (Szczecinski et al., 2021). A model whose values of *a, b, c, d*, and τ are tuned so that the model can reproduce the response to one stimulus can reproduce the response to different stimuli without retuning the parameter values (Szczecinski et al., 2021). The values used in this study can be found in Table 2. They are based on the responses of tibial CS. In future work, we will collect recordings from the trochanteral CS with which to tune the model.

**TABLE 2:** CS model parameters of groups 6B, 6A, 3, and 4.

Model parameters					
Group/parameter	a	b	c	d	τ
6B	338.9952	2.2707	7.1531	−27.9311	0.0250
6A	338.9952	2.2707	7.1531	−17.9311	0.0250
3	338.9952	2.2707	7.1531	−27.9311	0.0250
4	338.9952	2.2707	7.1531	−27.9311	0.0250

The controllable parameters for the model are a, b, c, d, and τ.

### 2.5. Treadmill and dynamic scaling

The variable speed treadmill is used to allow for movements that simulate the leg pushing the body forward. The linear guide forces the leg to support the guide’s weight during stance phase. The treadmill is synced to the leg actuators using a calibrated tachometer.

One key component of this experiment is that the robot is dynamically scaled to the insect. This means that it experiences a similar balance of inertial, viscous, elastic, and gravitational forces. Because the servos in the robotic leg affect its dynamics, the following scaling procedure ensures that the robot experiences (scaled) forces similar to what the insect would experience. To ensure the robotic leg is dynamically scaled to the insect’s leg, the robotic leg’s stepping cycle period needs to be the same proportion of its joints’ natural frequency of oscillation as in the animal. As a result, we approximate the natural frequency of the animal’s femur-tibia joint, compare it to the animal’s observed stepping cycle period, then use the natural frequency of the robotic leg’s femur-tibia joint to select an appropriate stepping cycle period for the robot.

The insect’s leg is approximately a slender rod with mass of 11 mg (Cruse and Bartling, 1995) and length of 1.2 cm, meaning its moment of inertia about its end is approximately $J_{O}=\frac{1}{3}\cdot mL^{2}5.310^{-10}kg\cdot m^{2}$. The stiffness of its femur tibia joint due to the passive elastic forces of its muscles is approximately $k_{T}=10^{-6}\frac{Nm}{rad}$ (Dallmann et al., 2017). Thus, the natural period of the femur tibia joint is about $T_{n}=\sqrt{k_{T}/J_{O}}=0.14s$. The duration of a stick insect step is on the order of 1 s, meaning that the duration of each step is approximately six times longer than the natural period. This means that the robot’s step period should be six times longer than its natural period of oscillation. Due to the 250:1 gearbox of the servomotor, the motor’s rotor is the main source of the inertia about the femur-tibia joint. This value is approximately *J*_*O*_ = 10^−2^*kg*⋅*m*^2^. The stiffness of the servo’s feedback controller is approximately $k_{T}=1\frac{Nm}{rad}$. The resulting natural period of oscillation of the femur-tibia joint is approximately $T_{n}=\sqrt{k_{T}/J_{O}}=0.63s$, and six times this value is approximately 4 s. As a result, the dynamically scaled step duration for the robotic leg is 4 s while a *C. morosus* step duration is approximately 1 s (Cruse and Bartling, 1995).

## 3. Results

The results of this study include several comparisons between data from animal experiments and from our robotic model. For clarity, we define the following terms. For biological experiments, “sensory discharge” is the resulting activity of the sensory neurons measured in action potentials per second. Such data has been collected by extracellular recordings of leg nerves while controlled forces are applied to insect legs (for example, Ridgel et al., 2000; Noah et al., 2001, 2004; Keller et al., 2007; Zill et al., 2009, 2021; Harris et al., 2022). Action potentials are identified in the recordings and then “binned” over time to approximate the discharge in action potentials per second. For robot experiments, the strain of the leg segment measured by the strain gauge is the “strain.” The output of the mathematical CS model in response to the robot leg strain is the “model discharge.” This model takes the strain signal as its input and produces a continuous output that models the approximate sensory discharge in action potentials per second.

### 3.1. Encoding of forces by leg strain gauges

**FIGURE 4:**
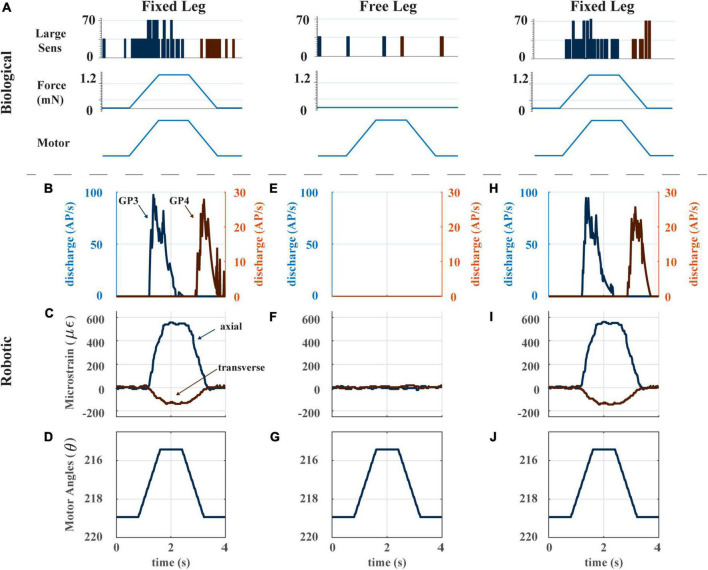
**(A)** Biological experiment with ramp and hold stimulus with resulting CS output. Adapted from Figure 6C of Zill et al. (2012). **(B)** The model CS discharge (group 3 and 4) in response to a single ramp-and-hold-and-release stimulus with distal end of the trochanterofemur fixed. **(C)** Axial and transverse strain of the trochanterofemur with distal end fixed. **(D)** Ramp-and-hold-and-release motion commanded to the Dynamixel servomotors. When the distal end of the trochanterofemur is fixed, the servo’s torque generates the bending moment that strains the segment. **(E)** The model CS discharge (group 3 and 4) in response to a single ramp-and-hold-and-release stimulus with distal end of the trochanterofemur free to move. **(F)** Axial and transverse strain of the trochanterofemur with distal end free. **(G)** Ramp-and-hold-and-release motion commanded to the Dynamixel servomotors. **(H)** The model CS discharge (group 3 and 4) in response to a single ramp-and-hold-and-release stimulus with distal end of the trochanterofemur fixed. **(I)** Axial and transverse strain of the trochanterofemur with distal end fixed. **(J)** Ramp-and-hold-and-release motion commanded to the Dynamixel servomotors.

Two features of CS sensory discharge that we wish for our robotic leg model are (1). Discharge occurs in response to imposed bending loads, not inertial forces from leg motion; and (2). Large CS caps produce phasic sensory discharge in response to imposed bending loads. Figure 4A illustrates these features using data from Zill et al. (2012). When the distal end of an insect’s leg segment (in these experiments, the tibia) is fixed and the leg joint is actuated by pulling on the muscle tendon with a ramp-and-hold-and-release stimulus, the sensory discharge of large CS on the leg signals the ramp portion of the stimulus and then adapts during the hold portion (Figure 4A, left). When the distal end of the leg segment is free, the CS discharge is effectively zero (Figure 4A, center). This indicates that the sensory discharge signals the strain on the leg segment due to bending imposed by external forces, not due to inertial forces arising from the insect’s motion. Fixing the distal end of the leg segment and repeating the experiment causes discharge to return, ensuring that the lack of activity in the unfixed case was not due to damage to the leg (Figure 4A, right). For our robot to be a model of insect strain sensing, its CS model discharge should share these properties.

**FIGURE 5:**
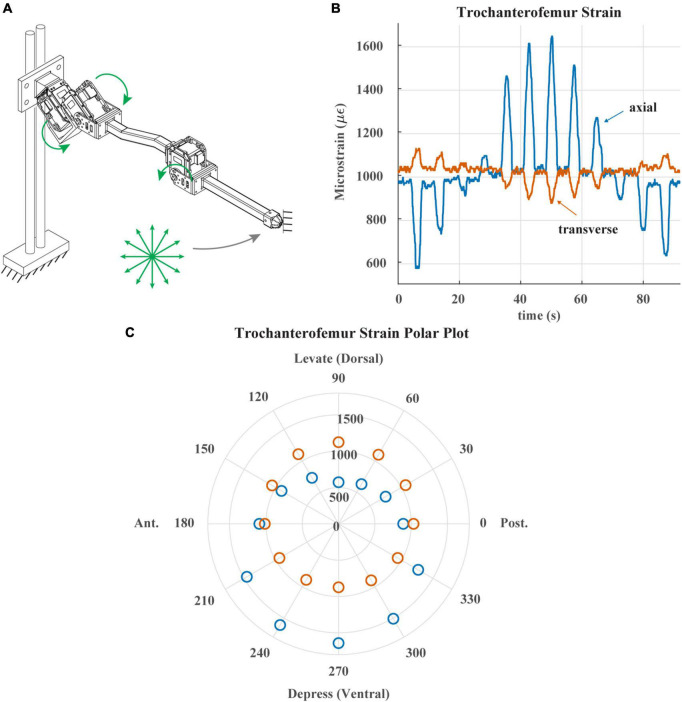
**(A)** Diagram of robotic leg configuration. Green arrows show type and direction of movement for the servomotors. Distal end of leg is fixed in place and the leg is commanded to draw a 12-point asterisk shown with green arrows. **(B)** Trochanterofemur strain output when distal end of leg is fixed, and the robotic leg is commanded to draw an asterisk pattern. The leg would return to the center of the asterisk before moving to the next position. **(C)** Polar plot of the trochanterofemur strain data with anterior, posterior, dorsal and ventral sides of robotic leg labeled.

**FIGURE 6:**
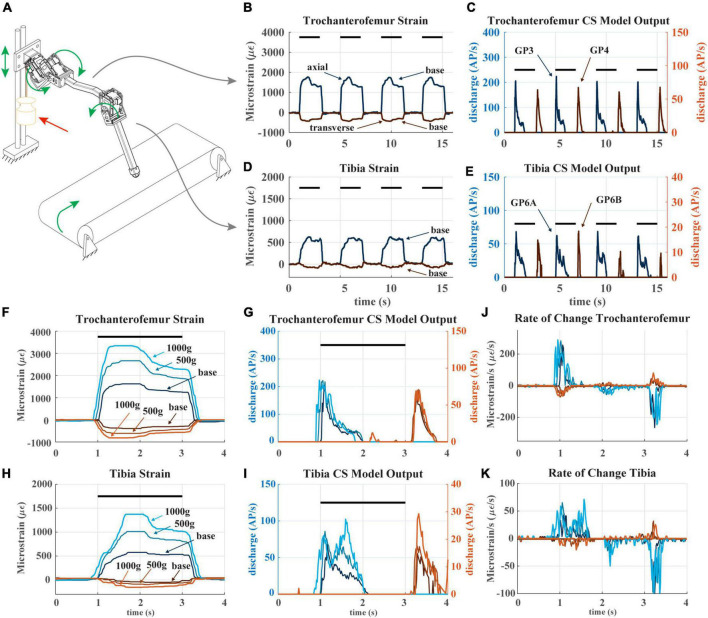
**(A)** Diagram of robotic leg configuration. Green arrows show type and direction of movement for the servomotors, linear guide, and treadmill. A weight indicated by the red arrow can be added to the linear guide to simulate additional body weight. **(B)** The raw trochanterofemur strain data of four subsequent steps in baseline configuration (no added weight). The axial strain is indicated in shades of blue and transverse indicated in shades of red. Positive changes in values indicate compression in that direction; negative changes in values indicate tension in that direction. The black bars indicate stance phase. **(C)** The trochanterofemur (group 3 and 4) CS model discharge from four subsequent steps in baseline configuration. **(D)** The tibial strain data from the same four subsequent steps **(E)**: The tibia CS model discharge (group 6B and 6A) from four subsequent steps in baseline configuration. **(F)** The raw trochanterofemur strain from individual steps in each of the three load configurations. The configurations are baseline (no added weight), 500 g configuration (added weight to linear guide), and 1,000 g configuration (added weight to linear guide). **(G)** The CS model discharge of the trochanterofemur groups in the three configurations. **(H)** The raw tibia strain of individual steps in the three configurations. **(I)** The CS model outputs of the tibia groups in the three configurations. **(J)** Rate of change of the trochanterofemur strain. **(K)** Rate of change of the tibia strain.

The robot’s CS model discharge captures the two features listed in the previous paragraph. Figures 4B–J shows the response of the robot’s CS sensors to a ramp-and-hold-and-release stimulus with the distal end of the leg fixed, with the distal end of the leg free, and then again with the distal end of the leg fixed. When the distal end of the leg is fixed, the strain reflects the bending moment imposed on the leg segment and shows a clear trapezoidal shape through time (Figures 4C, I). The model CS outputs (Groups 3 and 4) show clear indications of the start and end of the stimulus (Figures 4B, H). If the leg moves freely, the strain output is essentially zero (Figure 4F). Furthermore, this lack of strain is reflected in zero output of the CS model (group 3 and 4, Figure 4E). This result indicates that the strain in the leg segment is due to external forces and moments imposed by contact forces with the environment, not due to inertial forces generated by the motion of the leg. This result suggests that the motion of the robot has been dynamically scaled to match the insect, and that our robot’s model discharge may reflect the discharge experienced by the animal. A quantitative comparison of the model discharge to the animal sensory discharge was published in Table 3 in Szczecinski et al. (2021), which reports the mean absolute error between them (mean error < 10% for most trials, mean error 19.5%, error standard deviation 21.9%) (Szczecinski et al., 2021).

Figure 5 demonstrates the directionality of the strain gauges on each leg segment. Figure 5A illustrates the way in which the trochanterofemur segment was loaded. The ThC and CTr servos were commanded to draw an “asterisk” shape with the distal end of the trochanterofemur while the end of the leg was fixed in place, resulting in bending forces that cycled from dorsally directed to anteriorly directed to ventrally directed to posteriorly directed, separated by 30 degree increments. Figure 5B plots the axial and transverse trochanterofemoral strain as the segment was loaded. Figure 5C plots the average strain response during each hold phase in a polar arrangement, in which the angle is the orientation of the force and the radius is the amplitude of the strain. As seen in the stick insect (c.f., Figure 4D of Zill et al., 2012), Group 3 primarily responds to dorsally directed bending forces and Group 4 primarily responds to ventrally directed bending forces. Together, Figures 4, 5 demonstrate that the robotic model captures the basic temporal and spatial responses of CS to leg loading, which the following results build upon.

### 3.2. Effects of increased body load on force signals

To test how changes to body weight (e.g., due to insect growth, egg gestation, carrying objects) may affect the CS discharge an insect would experience while walking, we recorded robot strain and model the large CS discharge as the leg stepped on the treadmill and then added loads to the carriage device that emulated body weight. Figure 6A contains a diagram of how the robotic leg was utilized. The green arrows indicate how each object is permitted to move. In this experiment, all servomotors in the leg rotate, the “body” carriage on the linear guide can only move vertically, and the treadmill is powered, simulating the propulsion of the body by the other legs. The location of the weight applied to the linear guide is also shown.

Figures 6B–E plots the strain and model discharge of the trochanterofemoral and tibial CS groups during four subsequent steps with the robot in the “baseline configuration,” i.e., when no weight is added. Each of the robot’s steps was effectively identical to the others, resulting in nearly indistinguishable strain and CS model discharge from step to step. Note that the axial trochanterofemoral axial strain is the largest because the weight of the “body” produces a large bending moment due to this segment’s orientation. The trochanterofemoral axial CS model discharge (group 3) rapidly increases at the beginning of stance due to the rate-sensitive nature of CS discharge. The model of the large CS discharge adapts throughout the first half of stance and then is silenced about halfway through stance. Again, this is due to the rate-sensitive nature of CS discharge. The strain decreases at this point due to the changing orientation of the leg relative to gravity (see Figure 5). At the end of stance phase the trochanterofemoral transversal CS model discharge (group 4) increased. This is a rate-sensitive “rebound” effect that is also present in insect CS sensory discharge (Harris et al., 2022). A similar condition occurred with the tibial CS (group 6B, 6A) although on a smaller scale because the tibia experiences less strain due to its orientation (Theunissen et al., 2015). The model discharges strongly indicate the beginning and end of stance phase.

The sensitivity of CS model discharge to the amplitude of additional weight depended on the location of the CS on the leg. For comparison, Figures 6F–I plots strain and discharge from one step in each of the three configurations (i.e., no additional weight or “baseline,” 500 g added to carriage on linear slider, and 1,000 g added). For each sensor location, the shape of strain remains relatively consistent as mass is added, except that the amplitude increases (Figures 6F, H). However, the trochanterofemoral CS model discharge is insensitive to the additional body mass (Figure 6G), while the tibial CS model discharge changes substantially in response to the additional body mass (Figure 6G). This is likely due to the rate-sensitivity of the CS model (Szczecinski et al., 2021). For the trochanterofemoral CS, the strain’s rate of change is consistent across all three load configurations (Figure 6J), meaning that the model discharge hardly changes. In contrast, the rate of strain experienced by the tibial CS greatly depends on the load configuration (Figure 6K), in particular between t = 1.2 s and t = 1.8 s (Figures 6H, I). CS groups at all locations strongly indicate the beginning and end of stance phase. However, due to the way the strain on the tibia increases throughout stance, the tibial CS model discharge also reflects the amount of mass added to the “body.” Such information could be valuable in the control of a robot or insect.

### 3.3. Effects of transient load increases

**FIGURE 7:**
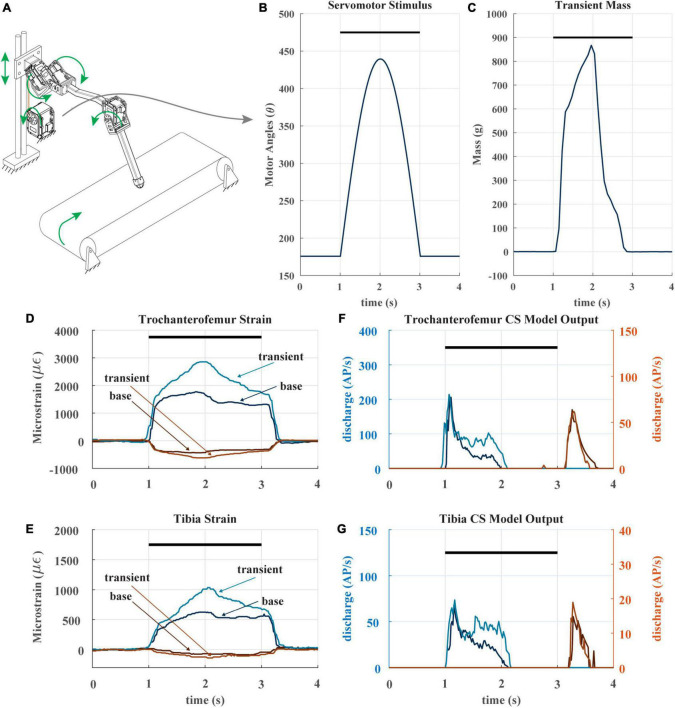
**(A)** Diagram of robotic leg configuration. Green arrows show type and direction of movement for the leg servomotors, perturbation servomotor, linear guide, and treadmill. The perturbation servomotor can apply a controlled load to the linear guide. **(B)** The applied angle positions of the perturbation servomotor. Black bar indicates stance phase **(C)** resulting transient mass to linear carriage from perturbation servomotor. **(D)** The raw trochanterofemur strain of individual steps in the baseline and perturbation configuration. Positive changes in values indicate compression in that direction; negative changes in values indicate tension in that direction. Black bar indicates stance phase. **(E)** The raw tibia strain of individual steps in the baseline and perturbation configuration. **(F)** The model CS outputs (group 3 and 4) of the trochanterofemur baseline and perturbation steps. **(G)** The model tibia CS (group 6B and 6A) outputs of the baseline and perturbation steps.

When animals and robots walk, a leg may suddenly be subjected to an increase in load it must support, for example, when an adjacent leg enters swing phase (Dallmann et al., 2017; Günzel et al., 2022). To determine whether CS discharge may reflect such increases, we subjected our robotic leg to a transient load in the middle of its stance phase. Figure 7A depicts the experiment, in which the linear guide is transiently pulled downward *via* cable attached to a spool and actuated by an additional servomotor. The servomotor applies a half sine-like force stimulus as seen in Figure 7B at the beginning of stance. The result of the stimulus is the transient mass seen in Figure 7C. The impact of this stimulus on the strain can be seen in Figures 7D, E. As noted previously, the CS model discharge is rate sensitive, because it is always trying to return to baseline. The transient load prolongs the period of increasing trochanterofemoral strain and increases the rate of change of the tibial strain, increasing the model discharge for the axial sensors between 1.5 and 2 s (Figures 7F, G). Such increased discharge could be used to produce compensatory motor output in an animal or robot.

### 3.4. Effects of leg slipping during walking

**FIGURE 8:**
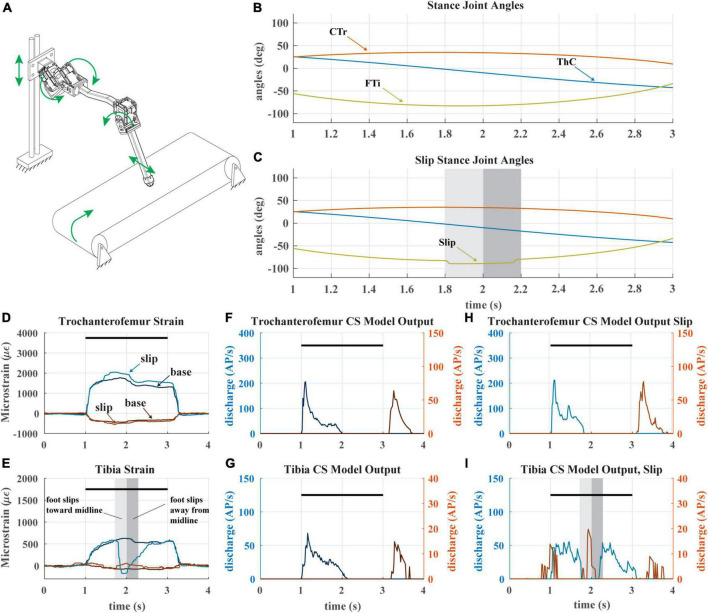
**(A)** Diagram of robotic leg configuration. Green arrows show type and direction of movement for the servomotors, linear guide, and treadmill. A green arrow shows the direction the tibia is forced to slip in. **(B)** Stance joint angles of servomotors in baseline configuration (no slip condition) **(C)** Stance joint angles of servomotors with added slip condition as seen in middle of stance. Shaded bars indicate duration and location of slip condition. **(D)** The raw trochanterofemur strain of individual steps in baseline and slip configurations. Positive changes in values indicate compression in that direction; negative changes in values indicate tension in that direction. The axial is indicated in shades of blue and transverse indicated in shades of red. The black bars indicate stance phase. **(E)** The raw tibia strain of individual steps in baseline and slip configurations. **(F)** The model CS output of the trochanterofemur in baseline configuration. **(G)** The model CS output of the tibia in the baseline configuration. **(H)** The model CS outputs of the trochanterofemur in slip configuration. **(I)** The model CS outputs of the tibia in the slip configuration.

A recent study hypothesized that the adaptive nature of CS sensory discharge may assist a walking animal in detecting tarsus slipping during stance phase (Harris et al., 2022). We tested this hypothesis with our robotic model leg by causing the tarsus to slip mid-stance as the leg stepped on the treadmill. Figure 8A depicts the experiment. The typical walking kinematics (Figure 8B) were modified such that the tibia flexes inward and then extends outward during stance phase, causing the foot to slip laterally across the treadmill surface (Figure 8C). When the tarsus breaks contact with the treadmill, the distal end of the tibia suddenly becomes free, and the tibia is no longer subjected to a bending moment.

During the slip, the sudden disappearance of the bending moment on the tibia causes the tibial strain to rapidly decrease to 0 (Figure 8E), which silences the axial (Group 6B) tibia CS model discharge and activates the transverse (Group 6A) tibia CS model (Figure 8I). This Group 6A discharge, which would normally occur at the end of stance as seen in Figure 8G, would be an unexpected signal that could trigger multiple motor responses, for example, reducing the activation of the tibia flexor muscle (Zill et al., 1981) or initiating swing phase to reposition the leg (Ridgel et al., 1999). When the direction of slip is reversed, the opposite trend is observed; the transverse strain decreases, which causes group 6B model discharge. The slip of the tarsus also affects the strain of the trochanterofemur (Figure 8D), which causes a phasic increase in the CS model discharge (Figure 8H) as compared to the baseline configuration (Figure 8F). The adaptive nature of CS feedback could make it very useful for detecting the slip of the tarsus across the substrate in both insects and robots.

## 4. Discussion

In this investigation, we compared encoding of forces in a robotic leg and to the middle leg of stick insects. Strain gauges were placed on the robotic leg in four locations and orientations corresponding to major groups of campaniform sensilla (CS) of the middle legs of stick insects: Groups 3, 4, 6A, and 6B. Experiments were performed on the mechanical leg that were similar to those used to characterize the responses of the biological receptors. After establishing that the robotic model’s force encoding was similar to that of the animal, we used the robotic model to generate hypothetical sensory discharge that may be produced *in vivo* as the animal walks. Perturbing the leg by adding constant and transient loads and causing its tarsus to slip provided insight into how CS in the animal may signal such conditions. This study can therefore provide unique insights into the types of force information that occur in stick insects and how this information could be used in control of a walking robot.

In the same way as insects’ CS discharge (Ridgel et al., 1999; Harris et al., 2022), the CS model discharge emphasizes the dynamic changes in force that occur at the beginning and end of stance phase. This is because the model discharge, like the sensilla discharge, is highly sensitive to the rate of change of force (Zill et al., 2021). Detecting the initiation and end of stance phase is critical to the coordination of walking in insects (Cruse, 1990; Duysens et al., 2000; Zill et al., 2004; Dallmann et al., 2017), underlining the importance of CS and their sensory discharge dynamics. We hypothesize that a walking robot that employs such filtering may have improved interleg coordination over one that does not. This will be explored in future work with closed loop control in a six-legged robot.

Insects appear to utilize CS sensory feedback in a number of ways. All leg CS are sensitive to force dynamics and tests using joint torques derived from freely walking animals (Dallmann et al., 2016) indicate that the discharges of the receptors reflect variations in the rate of change of forces rather than simply encoding the force level (Zill et al., 2021). These signals could be used to adjust muscle activities to compensate for force variations to ensure smooth movements in walking. For example, feedback from the tibial CS may adjust the motor output of the muscles in the leg to prevent tarsus slipping during walking (Zill et al., 1981). In contrast, feedback from the trochanteral CS may *enhance* muscle activity and rapidly generate support and propulsion at the start of the stance phase (Zill et al., 2012). These findings imply that the signals from the receptors are used flexibly depending upon the receptor location and specific behavior being performed. However, it may be difficult to confirm the role of such feedback directly, because of the difficulty of recording from so many sensilla simultaneously in a moving animal. The robotic leg we have presented in this study does not have such limitations, and as a result, may enable us to clarify the role of load feedback from across the leg by performing more experiments that would be impossible to conduct with an insect.

Although our experiments provide insight into the role of adaptive load sensing in locomotion, our study has several limitations. First, our single-leg robotic model does not capture all the dynamics of a freely standing, walking animal. It is known that the activity of other legs on the body can shape the force experienced by any one leg (Dallmann et al., 2017). Although we attempted to mimic this condition by mounting the leg to a vertically sliding carriage, the carriage’s rails prevent the “body” from moving laterally or anterior-posteriorly. We plan to repeat the experiments in this study with a freely walking robot in the future. Another limitation of our study was that our robot is much larger than an insect (15:1 scale) and its mass is distributed differently across the leg. We corrected for the difference in scale by slowing the motion of stepping, which reduced inertial forces in the leg and prevented leg strain due to vibration (Figure 3). Furthermore, the leg and carriage have a total mass of 800 g, and less than 100 g can move relative to the carriage, meaning that more than 87% of the mass is concentrated in the body. This figure is consistent with a locust, 83% of whose mass is concentrated in the body and the remaining 17% is concentrated in the legs (Bennet-Clark, 1975; Alexander RMcn., 1995). Thus, despite the difference in size, actuators, and materials between the insect and the robot, we expect that our results are generally applicable to the animal.

### 4.1. Comparison to biomechanics and neurophysiology

After filtering and processing, the signals from the strain gauges closely corresponded to those found in campaniform sensilla in the insect leg. The measured strain could be processed by the mathematical CS model to generate animal-like signals in response to both force increases and decreases (Figure 4). Like the biological receptors, strain gauges enable directional force reception (Figure 5). Furthermore, processing through the model could emulate the variable extent of adaptation found in the biological sensors, in particular, the dramatic adaptation of large receptors to tonic forces. Thus, the robotic leg successfully emulated some characteristics of stick insect campaniform sensilla discharge.

One defining note is that the model could reliably differentiate between the start and end of stance phase. This is shown in the model outputs as groups 3 and 6B increase amplitude at the start of stance and silence as the force decreases. Groups 4 and 6A show an opposite trend as they increase at the end of stance and silence after a short period of time. These results mimic CS as they have been described in insects (Zill et al., 2004; Keller et al., 2007). However, it is important to note that our model emulates the characteristics of the larger CS rather than the smaller CS, whose discharge adapts to a lesser degree than that of the larger CS (Zill et al., 2011). The animal sensory discharge in response to the ramp-and-hold-and-release shows more prolonged discharges and incomplete adaptations to sustained loads (Zill et al., 2011, 2012). Our model does not reproduce that effect for CS groups 3 and 6B. The model can be adjusted to react similarly, but it was not for this study. The model groups 4 and 6A discharge mimic the adaptation of the animal’s sensory discharge.

### 4.2. Applications in robotics

Our dynamic CS discharge model may facilitate the construction and operation of robots with many redundant sensors, like insects have. Robots often use as few sensors as possible, data from which are used to calculate the full state of the robot. This method is adopted for pragmatic reasons; configuring and calibrating sensors that operate reliably on board a robot is extremely challenging. However, this method increases the computational load placed on the control hardware, which may increase its power usage and consequently decrease robot runtime before recharging the batteries. Furthermore, this begs the question how small animals with limited resources, e.g., insects, can perform the complex calculations deemed necessary to control legged hardware. Insects (and subsequently robots) may reduce the complexity of calculations necessary for control by incorporating sensors that directly measure quantities of interest, e.g., the resultant force vector acting on the leg. As noted in the early study of Pringle (1938), insect CS may simplify control of the leg by signaling forces generated by groups of synergistic muscles (in contrast to measurements of the forces of individual muscles, as Golgi Tendon Organs do, or individual motors, as current sensors do). Although adding sensors to the robot may increase its complexity, our dynamic CS model may simplify the calibration of these sensors, due to its robust signaling of increases and decreases in load. It remains to be seen if our approach actually facilitates the processing of data from redundant robot sensors. In future work, we plan to apply this approach to hexapod robot, each of whose legs is instrumented like the robotic leg in this study.

More specifically, the interleg coordination of walking robots may be enhanced if load feedback were processed in a dynamic way. The interleg coordination of insects and other arthropods is known to depend on the load supported by each leg (Cruse, 1990; Ekeberg et al., 2004; Zill et al., 2004; Dallmann et al., 2017). Neuromechanical simulations (Ekeberg et al., 2004; Szczecinski et al., 2014; Goldsmith et al., 2020) and robotic models (Szczecinski et al., 2015; Dürr et al., 2019) of animal locomotion reinforce this notion. However, to the authors’ knowledge, no prior study has incorporated the dynamics of how CS measure load into their robot controller. Tuning strain sensors to eliminate false negative and false positive data about leg load is difficult. We hypothesize that the adaptive nature of the CS discharge model we have implemented will facilitate the accurate determination of when the leg is in swing or stance, which will ultimately improve interleg coordination in robots.

Intraleg coordination of robot leg control also stands to benefit from dynamic CS discharge modeling. Feedback from CS on one leg segment is known to affect the patterning of motor output that controls other leg segments (Akay et al., 2001). Robot controllers have mimicked this mechanism with either dynamic neural models (Goldsmith et al., 2020) or abstracted finite state machine controllers, in which the motion of each joint depends on load feedback from across the leg (Rutter et al., 2011). Dynamic CS discharge may serve such intraleg, intersegmental coordination by providing precisely timed, high-amplitude signals when the tarsus contacts the substrate and some joints should reverse their direction of travel. In future work, we will test whether this is true by embedding our CS model into the closed loop control of a walking robot.

### 4.3. Model robustness

The adaptive nature of the CS model makes the feedback robust to changes in loading. Much like insects, robots encounter many different and often unpredictable sensory stimuli. Sound decision making under uncertain conditions may be served by robust sensory feedback. Our experiments show that the CS model discharge at the beginning of stance is robust to changes in loading (e.g., trochanterofemoral discharge in Figure 6G. In all cases we tested, the model discharge clearly indicated the beginning and end of stance phase, suggesting that the adaptive strain processing performed by the CS model would increase the robustness of a control network that makes decisions about which joints to move at the beginning or end of stance.

Despite the robustness of the model discharge to changing load conditions, additional information about leg load was encoded in some cases. For example, when the foot slipped and the tibial strain rapidly dropped to 0, group 6A discharged, signaling that the tibia was no longer being strained (Figure 8I). Simultaneously, the trochanterofemoral strain increased. The adaptive nature of the CS model causes it to accentuate changes in load over time, signaling when the motor plan needs to be altered.

## Data availability statement

The raw data supporting the conclusions of this article will be made available by the authors, without undue reservation.

## Author contributions

NS and SZ conceptualized the study and contributed to engineering and biological expertise. WZ created the robotic leg and collected the data. All authors contributed to manuscript creation and revisions.
